# Cisplatin selects for stem-like cells in osteosarcoma by activating Notch signaling

**DOI:** 10.18632/oncotarget.8849

**Published:** 2016-04-20

**Authors:** Ling Yu, Zhengfu Fan, Shuo Fang, Jian Yang, Tian Gao, Bruno M. Simões, Rachel Eyre, Weichun Guo, Robert B. Clarke

**Affiliations:** ^1^ Department of Orthopedics, Renmin Hospital of Wuhan University, Wuhan, Hubei, China; ^2^ Department of Orthopedic Oncology, Key Laboratory of Carcinogenesis and Translational Research, Ministry of Education, Peking University Cancer Hospital & Institute, Beijing, China; ^3^ Breast Cancer Now Research Unit, Institute of Cancer Sciences, University of Manchester, Manchester, UK

**Keywords:** osteosarcoma, cancer stem-like cells, chemo-resistance, Notch signaling pathway

## Abstract

Notch signaling regulates normal stem cells and is also thought to regulate cancer stem cells (CSCs). Recent data indicate that Notch signaling plays a role in the development and progression of osteosarcoma, however the regulation of Notch in chemo-resistant stem-like cells has not yet been fully elucidated. In this study we generated cisplatin-resistant osteosarcoma cells by treating them with sub-lethal dose of cisplatin, sufficient to induce DNA damage responses. Cisplatin-resistant osteosarcoma cells exhibited lower proliferation, enhanced spheroid formation and more mesenchymal characteristics than cisplatin-sensitive cells, were enriched for Stro-1+/CD117+ cells and showed increased expression of stem cell-related genes. A similar effect was observed *in vivo*, and in addition *in vivo* tumorigenicity was enhanced during serial transplantation. Using several publicly available datasets, we identified that Notch expression was closely associated with osteosarcoma stem cells and chemotherapy resistance. We confirmed that cisplatin-induced enrichment of osteosarcoma stem cells was mediated through Notch signaling *in vitro*, and immunohistochemistry showed that cleaved Notch1 (NICD1) positive cells were significantly increased in a relapsed xenograft which had received cisplatin treatment. Furthermore, pretreatment with a γ-secretase inhibitor (GSI) to prevent Notch signalling inhibited cisplatin-enriched osteosarcoma stem cell activity *in vitro*, including Stro-1+/CD117+ double positive cells and spheroid formation capacity. The Notch inhibitor DAPT also prevented tumor recurrence in resistant xenograft tumors. Overall, our results show that cisplatin induces the enrichment of osteosarcoma stem-like cells through Notch signaling, and targeted inactivation of Notch may be useful for the elimination of CSCs and overcoming drug resistance.

## INTRODUCTION

Osteosarcoma is the most commonly diagnosed primary malignant bone tumor, with a peak in incidence occurring in the second decade of life [1, 2]. The introduction of adjuvant and neoadjuvant chemotherapies have greatly improved the long-term survival for osteosarcoma patients [3, 4], but chemo-resistance and recurrence remain common outcomes. Cisplatin is an effective antitumor agent with a wide spectrum of activity against solid tumors, and the inclusion of cisplatin in osteosarcoma treatment has improved outcome for patients with high grade disease [5]. Mechanistically, cisplatin exerts its antitumor effects predominantly through yielding DNA intra-strand cross links between adjacent purines. This results in inhibition of DNA replication, transcription, and ultimately leads to cell death [6, 7]. Some patients present with intrinsic or acquired resistant to cisplatin, leading to recurrence and metastasis. However, the underlying mechanisms of cisplatin resistance are still unknown [8].

In recent years, it has been proposed that cancers contain a sub population of cells called cancer stem cells (CSCs), which are responsible for the maintenance and growth of tumors [9]. A variety of techniques have been used to isolate CSCs from osteosarcoma. Gibbs et al were the first to show that a small population (0.1 -1%) of osteosarcoma cells formed spheres in serum-free low attachment conditions. These spheres had self-renewal ability as well as increased expression of the embryonic stem cell markers Oct4 and Nanog [10]. Adhikari et al. showed that CD117/Stro-1 double positive osteosarcoma cells possessed the stem cell-like properties of resistance to chemotherapeutic reagents, increased tumorigenicity, and increased capacity to metastasize *in vivo* [11]. Based on findings that osteosarcoma spheres had increased TERT expression, we engineered osteosarcoma cell lines that stably express a human TERT promoter-driven GFP reporter. These TERT/GFP+ cells showed enhanced stem cell-like properties both *in vitro* and *in vivo*, including tumor propagating capacity, metastatic activity and resistance to chemotherapeutic agents [12, 13]. However, the signaling pathways that regulate the osteosarcoma stem cell phenotype are unknown.

The Notch signaling pathway is evolutionarily conserved and regulates cell proliferation, survival, apoptosis and differentiation. Upon binding of a ligand (jagged 1, jagged 2, delta-like 1 or delta-like 1, 3 or 4) to the cell surface Notch receptors (Notch1-4), the intracellular domain of Notch (NICD) is cleaved and translocated to the nucleus to induce the expression of target genes including Hes1, Hes5, Hey1 and HeyL [14]. Dysfunction of the Notch signaling pathway may block differentiation and lead to malignant transformation [15–17]. Many alterations in Notch signaling are reported in osteosarcoma. For example, Tanaka et al revealed that Notch2, Jagged1, HEY1 and HEY2 were overexpressed in osteosarcoma biopsy specimens, and Notch pathway inhibition decreased the growth of osteosarcomas by regulation of the cell cycle [18]. Engin et al reported a significant up-regulation of Notch signaling in human osteosarcoma cell lines, osteosarcomas from p53 mutant mice and primary human osteosarcoma tumor samples, while Notch inhibition decreased osteosarcoma cell proliferation both *in vitro* and *in vivo* [19]. A role for Notch signaling in osteosarcoma was also identified by Tao et al, who conditionally expressed NICD in mouse immature osteoblasts and successfully induced the formation of bone tumors that displayed features of human osteosarcoma [20]. Despite this, the role of Notch signaling in osteosarcoma stem cells and chemotherapy response has not yet been elucidated.

The Notch signaling pathway participates in maintaining stem cells in the osteoblast lineage and may play a role in maintaining osteosarcoma stem cells [21, 22]. Therefore, this study aims to determine both the role of Notch signaling in osteosarcoma stem cells, and its contribution to cisplatin resistance.

## RESULTS

### Enrichment of chemo-resistant osteosarcoma cells *in vitro*

**Figure 1:**
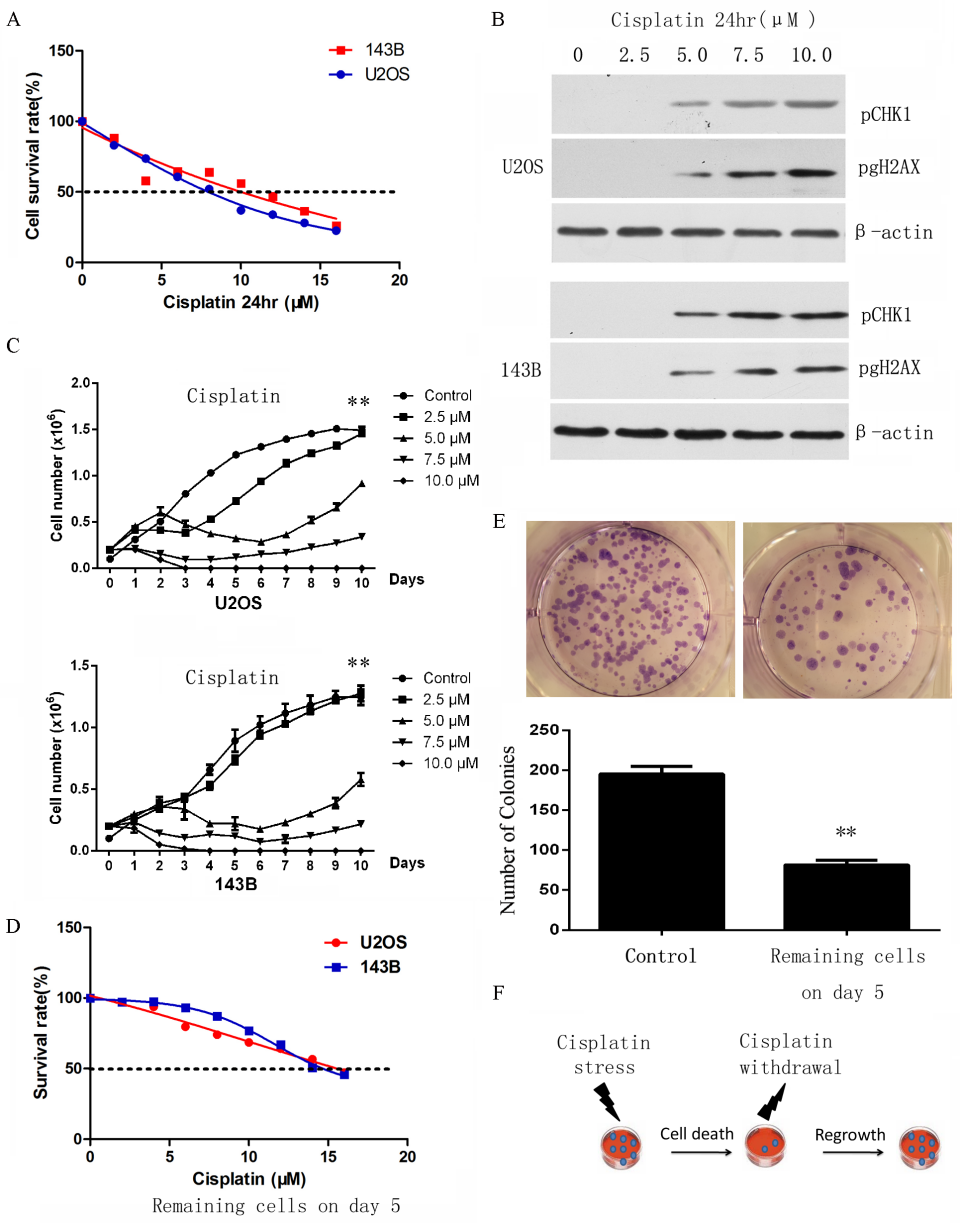
Selection of cisplatin resistant osteosarcoma cells. **A.** *In vitro* sensitivity of osteosarcoma cell lines to cisplatin as assessed by CCK8 toxicity assay. **B.** Activation of DNA damage response assessed by western-blot, confirming the effect of cisplatin. **C.** The growth of cells treated with short-term cisplatin was assessed by cell proliferation assay. p-values refer to 5.0 μM compared to control bars. **D.** The sensitivity of selected cells to cisplatin on day 5 was assessed by cell toxicity assay. Cells sensitivity to cisplatin was decreased in U2OS and 143B after 5 days of treatment. **E.** Colony formation assay on the selected osteosarcoma cells on day 5 demonstrated that remaining cells had a significantly lower clone number when compared with parental cells. **F.** Model illustrating the response of osteosarcoma cells to short-term treatment by cisplatin. Data are represented as mean ± SEM. *p < 0.05, **p < 0.01.

It is hypothesized that a small subset of cells with stem-like properties are resistant to chemotherapy. To determine if osteosarcoma stem cells are enriched upon chemotherapy, an *in vitro* chemoresistance model was established to mimic the heterogeneity observed in clinical settings. We first tested the cytotoxic effect of cisplatin in osteosarcoma cell lines to select a sub-lethal dose of cisplatin, which is sufficient to induce DNA damage. The osteosarcoma cell lines 143B and U2OS were treated with different concentrations of cisplatin for 24 hours, and cell viability and toxicity were determined by CCK8 assay (Figure 1A). The effect of cisplatin was confirmed by the activation of the DNA damage sensor phospho-gH2AX as well as the transducer phospho-CHK1 (Figure 1B).

The cytotoxic analysis results showed the IC50 for U2OS and 143B were 8.94μM (95%CI: 8.278 to 9.62) and 10.48μM (95%CI: 9.19 to 11.88) respectively. 2.5μM cisplatin did not induce DNA damage response (Figure 1B) whereas 7.5μM cisplatin induced significant cell apoptosis (Figure 1C). Therefore, 143B and U2OS cells were treated with 5μmol/L cisplatin for 24 hours, which is sufficient to induce DNA damage responses but not significant cell death, for the subsequent experiments. Cell growth after exposure to 5uM cisplatin for 24 hours was recorded for 9 days. In this time period cells suffered a short period of inhibition followed by a recovery from day 6 onwards (Figure 1C). The surviving cells of the U2OS and 143B cell lines on day 5 exhibited an IC50 of 15.66μM (95%CI: 14.87 to 16.52) and 16.17μM (95%CI: 14.75 to 17.87) respectively (Figure 1D), which is significantly higher than parental cells (*P* <0.01). Colony formation assay demonstrated that surviving cells on day 5 had a significantly lower colony number (Figure 1E), and flow cytometry showed these cells also had a significantly lower ratio of G2/M phase compared to mock cells (Supplementary Figure S1). These data indicate that the cells generated are low-proliferating resistant cells. Surviving cells at day 5 were thus used for subsequent experiments (Figure 1F).

### Cisplatin resistant osteosarcoma cells display characteristics of stem-like cells

**Figure 2:**
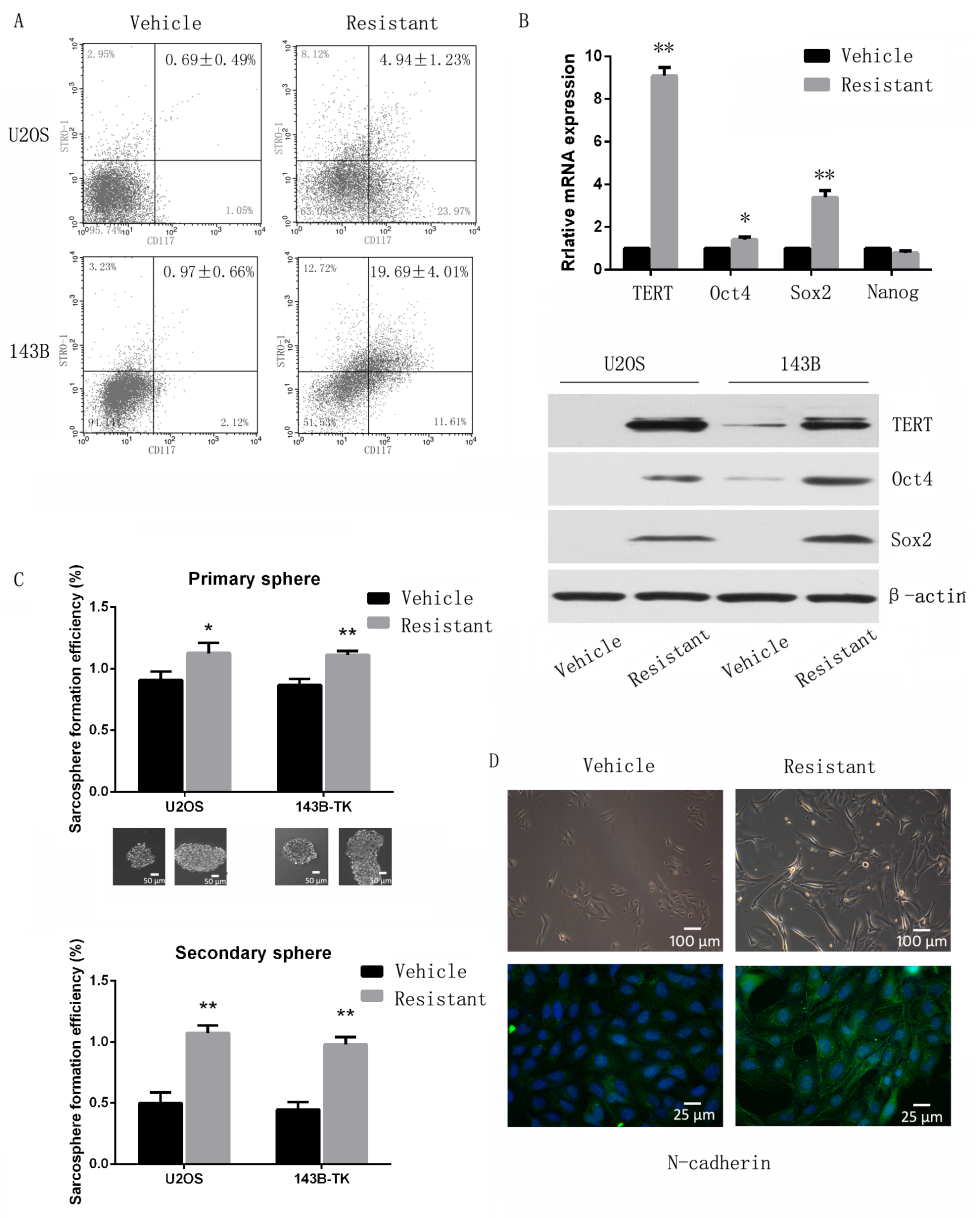
Chemoresistant osteosarcoma cells possess traits of cancer stem cells. **A.** Flow cytometry analysis of resistant and parental cells. The ratio of Stro-1/CD117 double positive cells was increased in resistant cells compared to vehicle cells. **B.** Stem cell-related genes were upregulated in resistant cells compared to vehicle cells when assessed by quantitative PCR and Western-blot. **C.** *In vitro* sphere forming self-renewal ability was enhanced in resistant cells. Secondary spheres also demonstrated enhanced serial sphere-forming capacity in resistant cells. **D.** Chemoresistant cells exhibit a more mesenchymal appearance, and immunofluorescence showed that N-cadherin was highly expressed in cisplatin resistance cells, indicating an EMT phenotype. Data are represented as mean ± SEM. *p < 0.05, **p < 0.01.

We next studied whether cisplatin-resistant osteosarcoma cells are enriched for CSCs. Cell surface markers have been reported for identifying osteosarcoma stem cells [11]. As shown in Figure 2A, cisplatin-resistant osteosarcoma cells showed an increased percentage of CD117/Stro-1 positive cells (*P* <0.01). Furthermore, the stem cell-related genes Oct4, Sox2 and TERT were upregulated in cisplatin-resistant cells (Figure 2B), and cisplatin resistant cells were able to generate more tumor spheres than vehicle cells during primary and secondary sphere assay (Figure 2C). Next, we tested whether cisplatin treatment could induce epithelial-mesenchymal transition (EMT). Immunofluorescence showed that N-cadherin was highly expressed in cisplatin resistant cells (Figure 2D), and EMT-TFs including Snail and Slug were also overexpressed in cisplatin resistant cells (Supplementary Figure S2).

Finally, we performed a limiting dilution assay to calculate TIC frequency. As shown in Table 1, 7/8 mice injected with 5×10^4^ 143B resistant cells formed tumors, whereas only 2 in 8 mice injected with 5×10^4^ vehicle cells formed tumors. The extreme limiting dilution assay (ELDA) calculation estimated a 17-fold increase in cancer stem cell frequency in resistant compared to vehicle cells. Collectively, these results indicate that cisplatin-resistant cells display stem cell-like properties *in vitro*.

**Table 1:** Tumor forming ability following subcutaneous injections

Cell number (143B)	Vehicle	Resistant	*P*-value
5,000	0/8	0/8	
50,000	2/8	7/8	
500,000	4/8	8/8	
ELDA	1/539493	1/31507	0.000
(95% CI)_a_	(1/1265641-1/229965)	(1/68568-1/14477)	

ELDA: Extreme limiting dilution analysis; CI: confidence interval.

### Osteosarcoma stem cells are enriched by cisplatin in a xenograft model

**Figure 3:**
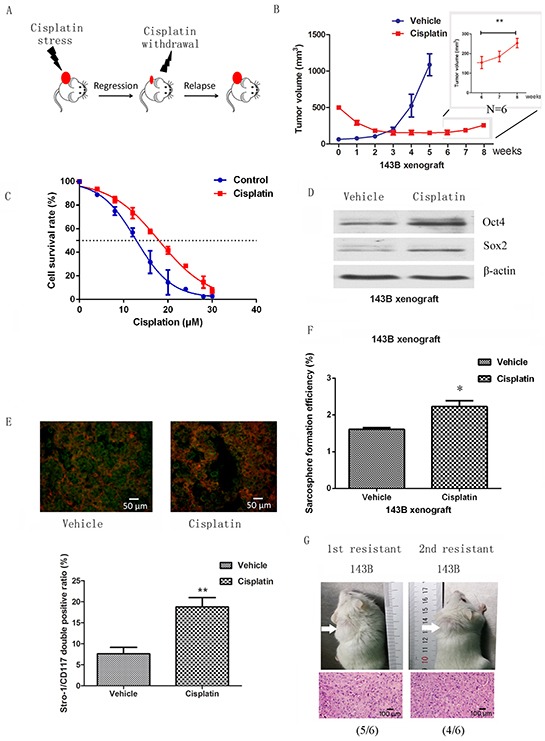
Cancer stem cells are enriched in a chemoresistant xenograft model **A.** Model illustrating the response of osteosarcoma xenograft to cisplatin. **B.** NOD/SCID mice with xenograft tumors of 10 mm in diameter were given 5 mg/kg of cisplatin at embedded intervals of 4 days for 4 weeks. Xenograft tumors of 5 mm in diameter were used as controls. A regrowth was observed after withdrawal of cisplatin for two weeks indicating that the majority of cells remaining in the small tumors were insensitive to cisplatin. **C.** Cells derived from cisplatin treated xenografts showed higher resistance to cisplatin. **D.** Stem cell-related genes were upregulated in resistant cells compared to vehicle cells when assessed by western blot. **E.** Stro-1/CD117 double positive cells were increased in resistance xenografts when assessed by immunofluorescence. **F.** Sphere forming assays showed that self-renewal ability was enhanced in cisplatin-resistant xenografts compared to cisplatin-sensitive. **G.** 5 × 10^4^ cells from cisplatin-resistant tumours were able to serially form tumors, whereas cells from the cisplatin-sensitive tumours were not. Data are represented as mean ± SEM. *p < 0.05, **p < 0.01.

We next enriched for osteosarcoma stem cells by establishing chemoresistant xenograft tumors in immunocompromised mice, mimicking the clinical situation under which osteosarcoma patients receive chemotherapy (Figure 3A). Tumor xenografts derived from 143B cells were subcutaneously inoculated into NOD/SCID mice. Mice bearing tumors of 10 mm in diameter (500 mm^3^ in volume) (n=6) were injected intraperitoneally with 5 mg/kg cisplatin at intervals of 4 days for 4 weeks. Vehicle treated cells grew rapidly and reached 1000 mm^3^ after five weeks, despite being implanted at 5mm diameter (62.5 mm^3^). In the cisplatin treated xenograft, the tumor mass decreased by 36.5% for the first two weeks and remained static for the next two weeks (Figure 3B). The remaining tumors were dissociated and tested for cisplatin sensitivity; these cells had an IC50 of 13.6 (95%CI: 11.9μM to 15.3μM), which is significantly higher than the parental cells (IC50 17.5μM, 95%CI: 16.6 to 18.4μM) (Figure 3C). In accordance with the *in vitro* results, tumors regrew following the withdrawal of cisplatin for 2 weeks (significant difference by week 8) (Figure 3B).

To confirm whether the proportion of CSCs in the residual chemoresistant tumor was increased following treatment, osteosarcoma cells derived from untreated (vehicle) and chemoresistant residual (chemoresistant) tumors were studied. Expression of stem cell-related genes, including Oct4 and Sox2, were dramatically higher in the chemoresistant group than the vehicle group (Figure 3D). We also found that Stro-1/CD117 positive cells were 7.6±2.6% in vehicle cells, compared to 18.7±3.9% in resistant xenografts (Figure 3E). In addition, cell sphere formation capacity was enhanced upon cisplatin treatment (Figure 3F). Cells from chemoresistant and vehicle xenografts were dissociated and tested for tumorigenicity. Tumorigenicity was examined after serial transplantation of corresponding cells into NOD/SCID mice by subcutaneous injection. 5 × 10^4^ cells from the resistant group were sufficient to form primary (5/6) and secondary tumors (4/6), whereas no tumors were formed from the vehicle group (0/6), indicating enhanced tumor-forming and self-renewal abilities in chemoresistant residual osteosarcoma cells (Figure 3G).

### Notch signaling regulates cisplatin-resistant CSCs

**Figure 4:**
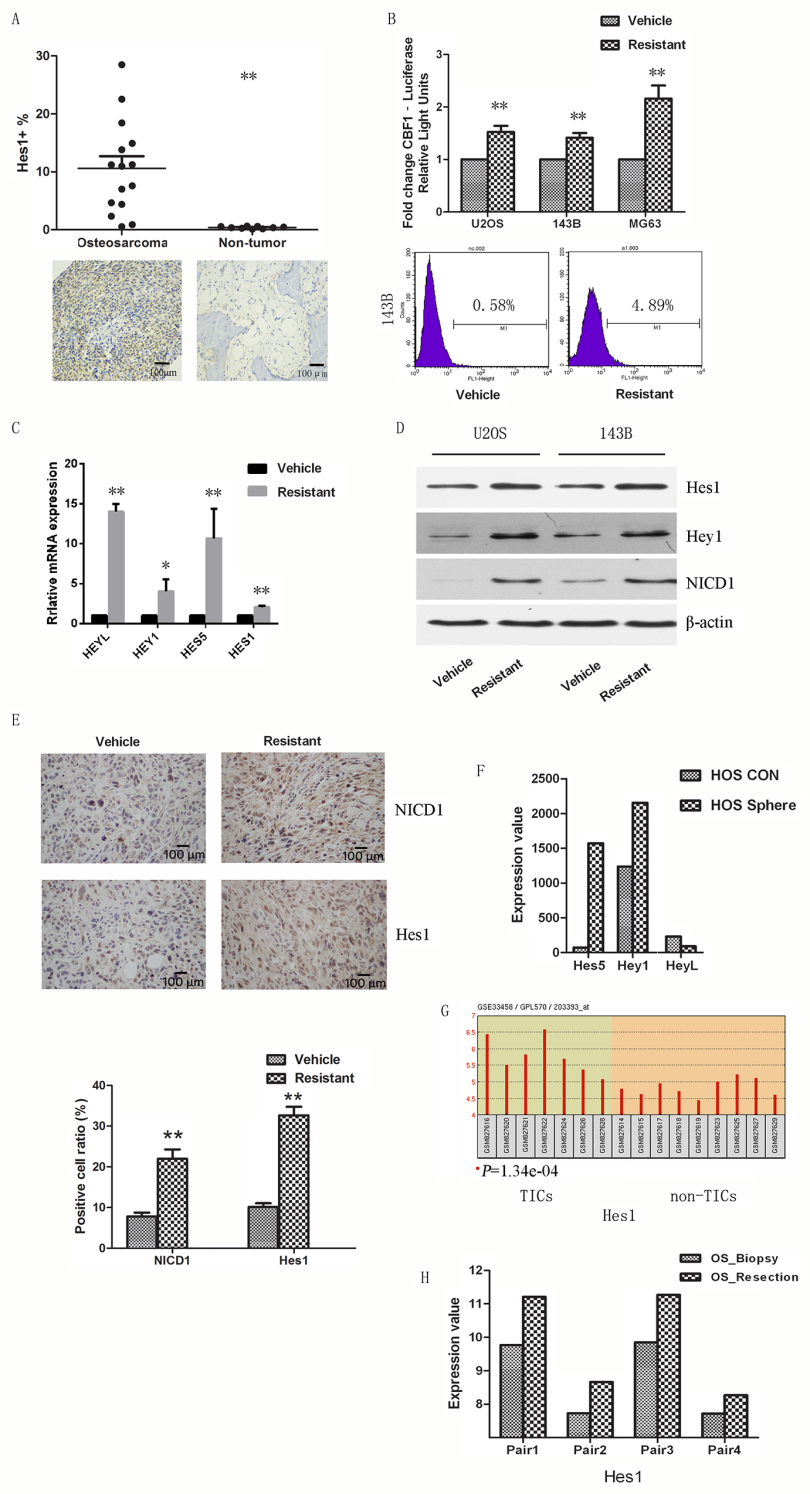
Notch signaling participates in cisplatin-resistant CSCs **A.** Hes1 was overexpressed in osteosarcoma specimens compared to matched non-tumor counterparts. **B.** Cells were transfected with lentiviral Notch reporter vector pGreenFire. Notch transcriptional activity was enhanced in cisplatin-resistant cells compared to parental cells by both luciferase assay and flow cytometry analysis. **C.** Notch target genes were upregulated in resistant cells compared to parental cells when assessed by quantitative PCR. **D.** Notch target genes and NICD1 were overexpressed in resistant cells compared to vehicle cells when assessed by Western-blot. **E.** Paraffin-embedded tissues of the xenotransplanted tumors were processed for IHC to detect NICD1 and Hes1 expression. The Notch pathway was activated in resistant xenografts, representative images presented. Several publicly available datasets were downloaded and analyzed. **F.** Notch genes were upregulated in MNNG/HOS sarcospheres compared to adherent control cells. **G.** In an *in vivo* study, Notch genes were increased in osteosarcoma initiating cells. **H.** Notch genes were also increased in resection samples compared to biopsy samples. Data were represented as mean ± SEM. *p < 0.05, **p < 0.01.

As the Notch signaling pathway regulates proliferation and differentiation of the osteoblast lineage, we sought to investigate whether the increase in CSCs by cisplatin treatment is regulated by Notch signaling in osteosarcoma cells. We first tested if the Notch pathway is activated in osteosarcoma specimens. We performed IHC staining for Hes1 on 15 human osteosarcoma tissue samples and their non-tumor counterparts. The percentage of Hes1+ cells in the osteosarcoma specimens ranged from 2.3% to 28.5%, whereas there was only 0.55% Hes1 expression in the non-tumor counterparts, mainly in multinucleated cells. Among all specimens, more than half (8/15) of the cases showed Hes1 expression in more than 10% of cells (Figure 4A).

We then tested the Notch transcriptional activity using a CBF1 reporter. Resistant cells had enhanced luciferase activity and GFP positive ratio compared with parental cells (Figure 4B). We examined the expression of Notch target genes by qPCR. Resistant cells had increased expression of Notch target genes, including Hes-1, Hes5, Hey1 and HeyL (Figure 4C). In agreement with the mRNA expression, protein levels of Hes-1 and Hey1 were also upregulated in resistant cells (Figure 4D). The level of cleaved Notch1 (NICD1) was also increased by cisplatin treatment, suggesting that Notch1 activation may play a critical role on cisplatin-induced enrichment of CSCs. Consistent with the results from the *in vitro* study, immunohistochemistry showed that NICD1 and Hes1 positive cells were 21.9% and 32.6% in the resistant xenografts (n=6) which had received cisplatin treatment, compared to 7.7% and 10.2% in the vehicle group (Figure 4E).

To further validate the relationship between Notch and osteosarcoma stem cells and chemoresistance, several publicly available datasets were analyzed [23]. *In vitro* results showed that Notch target genes were upregulated in MNNG/HOS sarcospheres compared with adherent controls (Figure 4F). Furthermore, an *in vivo* study using osteosarcoma cell derived xenografts showed that Notch target genes were increased in osteosarcoma initiating cells (Figure 4G). In a study that recruited biopsy/resection pairs, we also found that Notch target genes were significantly increased in resection samples compared to biopsy ones (Figure 4H). Notch1 receptor was also found to be highly expressed in these datasets (Supplementary Figure S3).

### Targeted inactivation of Notch signaling eliminates CSCs and overcomes drug resistance

**Figure 5:**
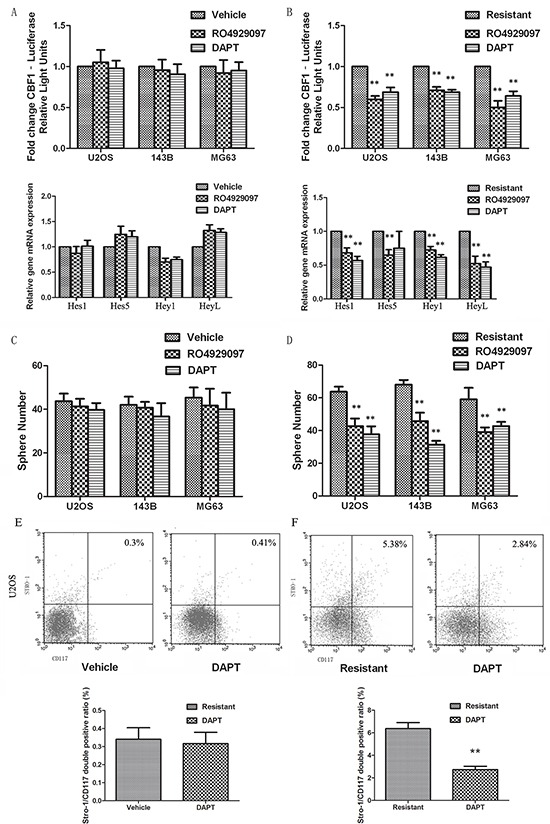
Notch inhibition eliminates CSCs and overcomes drug resistance **A.** and **B.** RO4929097 and DAPT had little effect on the Notch activity in vehicle cells, whilst significantly decreasing Notch activity in resistant cells. **C.** and **D.** Pretreatment with GSIs significantly inhibited cisplatin-induced sarcosphere formation, whilst no effect was seen in vehicle treated cells. **E.** and **F.** Pretreatment of GSIs inhibited cisplatin-induced enrichment of Stro-1/CD117 cells, whilst no effect was seen in vehicle treated cells. Data were represented as mean ± SEM. p-values refer to comparing to resistant group.*p < 0.05, **p < 0.01.

To investigate whether targeted inhibition of the Notch signaling pathway could reverse cisplatin resistance and decrease osteosarcoma stem cells, we treated cells with γ-secretase inhibitors (GSIs). RO4929097 and DAPT (20μM) did not alter Notch transcriptional activity and target gene expression in vehicle cells, but treatment decreased Notch activity by almost half in resistant cells (Figure 5A and 5B). Pretreatment with GSIs alone did not significantly alter sphere formation capacity or the ratio of CD117/Stro-1 double positive cells compared to control treatment. In contrast, pretreatment with GSIs (20μM) significantly inhibited cisplatin-induced sarcosphere formation (Figures 5C and 5D), enrichment of Stro-1/CD117 cells (Figures 5E and 5F), and expression of stem-like cell markers, including Oct4 and Sox2 (Supplementary Figure S4A).

**Figure 6:**
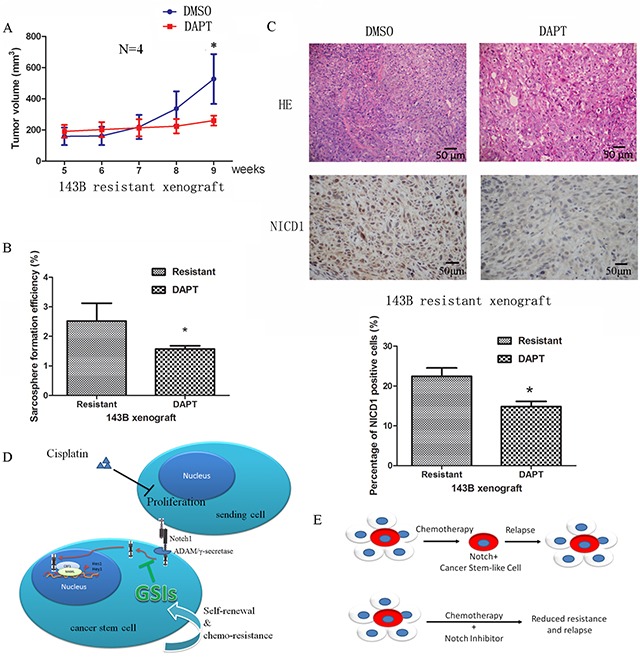
Notch signaling as a driving force of CSCs and chemoresistance **A.** Treatment with DAPT prevented tumour regrowth after cisplatin withdrawal *in vivo*. **B.** DAPT inhibited the sarcosphere formation capacity of cells from resistant xenografts. **C.** Paraffin-embedded tissues of the xenotransplanted tumors were processed for H&E and IHC staining. Representative images are presented showing that the Notch pathway was inactivated after treatment with DAPT. **D.** A schematic view depicting Notch signaling as a driving force of CSCs and chemoresistance. **E.** Diagram illustrating that chemotherapy does not target osteosarcoma stem cells, emphasizing the need to target residual drug-resistant cells to eliminate all cancer cells. Data were represented as mean ± SEM. *p < 0.05, **p < 0.01.

We then evaluated the effect of GSIs on the regulation of cisplatin-induced CSCs and resistance *in vivo*. Chemoresistant xenografts were treated with DAPT (Figure 6A), which prevented *in vivo* regrowth after cisplatin clearance. *In vivo* DAPT treatment of chemoresistant xenografts decreased sarcosphere formation *ex vivo* from 2.9±0.5% to 1.5±0.2% (*P* <0.05) (Figure 6B). The expression of stem-like cell markers were also decreased by DAPT treatment *in vivo* (Supplementary Figure S4B).

Finally, NICD1 expression was analyzed and shown to decrease from 22.4±4.2% to 14.8±2.5% after DAPT administration (*P* <0.01) (Figure 5C), confirming that the inhibitory effect was via the Notch pathway.

## DISCUSSION

Neoadjuvant chemotherapy in osteosarcoma leads to tumor regression, but is frequently followed by resistance [24]. So far, signaling pathways activated by cisplatin treatment have not been fully elucidated. Exposure to cisplatin elicits both pro-survival and pro-apoptotic signals, depending on the cellular contexts and the degree of DNA damage [25]. Here, we have selected a subpopulation of osteosarcoma cells which are resistant to cisplatin. We confirmed the effect of cisplatin on DNA damage by observing the phosphorylation of histone γ-H2AX foci and transducer phospho-CHK1[7]. Surviving cells suffered a short period of growth inhibition followed by a recovery. These cells were shown to have decreased proliferative capacity in addition to cisplatin resistance. We also established an *in vivo* model of chemoresistance. In this model short-term cisplatin treatment shrunk the tumor mass by 70% but enriched for chemoresistant cells, and was followed by tumour re-growth. We therefore successfully established both an *in vitro* and *in vivo* osteosarcoma cisplatin resistance model.

It is well established that CSCs are more resistant to standard cancer chemotherapies than bulk tumour cells. We and others have indicated that osteosarcoma stem cells are both tumour initiating and chemoresistant. [26–30]. In the present study, we provide direct evidence that cisplatin-resistant osteosarcoma cells possess stem cell-like properties. We confirmed the stem cell-like characteristics of resistant cells by detecting both the cell surface marker CD117/Stro-1 and stemness-related genes. We also tested the stemness of resistant cells by functional assays, which include spheroid formation and *in vivo* engraftment capacity.

Recent studies have linked EMT with the acquisition of stem cell-like characteristics [31–33]. For example, in breast cancer the EMT state has been associated with cancer stem cell properties including the expression of stem cell-associated CD44+/CD24-/low antigenic profile, self-renewal capabilities and resistance to conventional therapies [34–36]. Our previous study has shown that mesenchymal-derived osteosarcoma did not express epithelial markers, however, osteosarcoma stem cells express more mesenchymal markers and showed higher motility [13]. Consistent with this, we showed that resistant cells exhibited an altered morphology with increased long/short axis ratio and elevated expression of mesenchymal markers.

Previous data also implies that Notch signaling is involved in cisplatin-induced enrichment of CSCs [37]. Notch is a conserved pathway that has been implicated in the maintenance of tissue homeostasis by regulation of self-renewal and cell-fate determination in normal stem cells and early progenitors [38]. Recent studies have found that the Notch pathway is malfunctioned in osteosarcoma [18–20]. Our data showed that the Notch signaling pathway, and Notch1 in particular, plays a key role in the maintenance of osteosarcoma stem cells and cisplatin chemoresistance. We found that intercellular domains of Notch receptors, especially NICD1, were increased in chemoresistant cells, suggesting increased Notch activation. GSIs are commonly used as Notch pathway specific inhibitors, and various studies have confirmed the feasibility of using GSIs for targeting cancer stem cells [39–42]. In the present study, when the Notch pathway was blocked by GSIs we observed a significant depletion in sphere formation cells. Similarly, inhibition of the Notch pathway sensitized osteosarcoma cells to cisplatin treatment *in vivo*, further validating our hypothesis that the activation of Notch is necessary for the maintenance of osteosarcoma stem cells and cisplatin chemoresistance.

The relationship between cisplatin responses and Notch signaling is unclear. It has recently been reported that Notch negatively regulates the DNA-damage response, by binding to and inactivating ATM kinase. [43]. These data suggest that cisplatin removes cisplatin-sensitive cells and selects for cisplatin-resistant Notch positive cells. Other studies have shown that p53 is located upstream of Notch1, and Notch1 exerts a p53-dependent protective function against DNA damage through suppression of FOXO3 [44]. These data imply that p53 may be an intermediate between cisplatin effects and the activation of Notch signaling pathway.

Together, our studies show that cisplatin-resistant osteosarcoma cells possess stem cell-like properties and activated Notch signaling (Figure 6D). The data partially explain the high recurrence rate and chemo-resistance in patients receiving cisplatin-based treatment. Based on our results, we believe that inhibiting the Notch pathway could be a potential strategy in targeting osteosarcoma stem cells and overcoming cisplatin chemoresistance in a clinical setting (Figure 6E).

## MATERIALS AND METHODS

### Clinical specimens

15 patients with osteosarcoma were enrolled in this study. Patients were diagnosed between January 2009 and December 2011 at the Department of Orthopedic Oncology, Peking University Cancer Hospital & Institute with approval of Institutional Review Board (2015YW12). These patients received combination chemotherapy of cisplatin, methotrexate and doxorubicin for 2 to 4 cycles according to the guideline of NCCN (National Comprehensive Cancer Network). We obtained either the biopsy (before neoadjuvant chemotherapy) or resected (after neoadjuvant chemotherapy) tumur samples, clinicopathological features are listed in Supplementary Table S1. Samples of each patient were paraffin-embedded, sectioned, and used for evaluating Hes1 positive cells by immunohistochemistry.

### Publicly datasets and analysis

Several publicly available datasets (GSE38135, GSE33458, GSE39057, GEO2R) were analyzed to assess the potential role of Notch in osteosarcoma stem cells and chemotherapeutic resistance [23]. GSE38135 contains expression data from the osteosarcoma cell line MNNG/HOS that has undergone either sphere culture or normal cell culture. GSE33458 contains gene expression analysis from PKH26Hi cells (TICs) and PKH26Lo cells (non-TICs) obtained from five orthotopic bone tumors generated by MNNG/HOS and 143B cells, and another two secondary lung metastasis to osteosarcomas are included in the analysis. GSE39057 contains gene expression data from 5 unique pairs of diagnostic biopsy and surgical resection specimens. GEO2R was used to compare two or more groups of samples in order to identify genes that are differentially expressed across experimental conditions.

### Cell culture

The human osteosarcoma cell lines 143B, U2OS and MG63 were obtained from China Centre for Type Culture Collection (CCTCC) (Wuhan, China). 143B cells were cultured in α-MEM containing 10% fetal bovine serum (FBS) and 1% antibiotics (penicillin 100 U/ml, streptomycin 100 μg/ml). U2OS and MG63 cells were cultured in DMEM containing 10% FBS and 1% antibiotics. Cells were propagated in a humidified environment at 37°C with 5% CO_2_ and 100% humidity. Cell viability was determined using Trypan blue staining. Culture medium was replaced every three days.

### Generation of stably transfected cell lines

The pGreenFire1-Notch-EF1-Puro lentiviral reporter vector (TR020PA-P) and packaging vectors were purchased from SBI (System Bioscience, Mountain View, CA). HEK293-T cells were transfected with the constructs and viral supernatants obtained. To generate stably expressing reporter cell lines, osteosarcoma cells were infected with the viral supernatant and cells selected with puromycin (5μg/ml).

### Cell proliferation and cytotoxicity assay

The cells in each well containing 100 μl medium were incubated with 10 μl cell counting kit-8 (CCK-8) at 37°C for 2 h. The optical density (OD) of each well was then measured at 450 nm using a microplate reader.

### Clonogenic assay

Cells were seeded at 1000 cells per 60 mm culture dish and culture medium replaced every three days. After 7 days, the colonies were fixed with methanol and stained with 1% methylene blue solution. The colonies were determined by inverted microscope and colonies with a diameter ≥ 50μm were scored.

### Tumor spheroid assay

Cells were plated in ultra-low attachment plates at a density of 2,500 cells/ml in RPMI 1640 supplemented with B27 supplement, 20 ng/ml human epidermal growth factor (EGF) and 20 ng/ml human basic fibroblast growth factor (bFGF). Following culture for 2 weeks, colonies with a diameter ≥ 50μm were regarded as sarcospheres and quantified by inverted phase contrast microscopy. Spheres were dissociated and re-plated to form the next generation of spheres every 14 days.

### FACScan analysis

To study Notch activity in individual cells, stably transfected Notch reporter cell lines were constructed, and cells were dissociated to single cells to detect the ratio of GFP positive cells. Cells were harvested and resuspended in 70% alcohol, then fixed at 4°C for 30min. After washing in PBS, cells were incubated with fluorescein labelled antibody (STRO-1 and CD117) or isotype control IgG at 4°C for 30min. Cells were washed again before analysis using a BD FACScaliber flow cytometer (BD Biosciences). The fluorescent intensities were analyzed with Cell Quest Software (BD Biosciences).

### Luciferase assay

Cells were transfected with Notch pGreenFire-CBF1 reporter (Systembio) and CMV-Renilla luciferase reporter. Plasmids were incubated with Lipofectamine 2000 at a ratio of 3:1 in OptiMEM for 15 minutes then the mix was added to the culture media for 48 hours. Cells were lysed and luminescence was assayed with the Dual-Glo Luciferase assay system according to manufacturer's instructions. Luminescence of the firefly luciferase was normalized to that of the renilla luciferase.

### Animals and transplantation assay

To determine the tumorigenicity *in vivo*, 6 weeks old NOD/SCID mice were purchased from and maintained at the Wuhan University Center for Animal Experiments. The care and use of animals has been reviewed and approved by the Institutional Animal Care and Use Committee (IACUC) (approval number: S01315022l).

For extreme limiting dilution assay (ELDA), live cells were counted by trypan blue staining, and suspended in 10μL of 50% Matrigel/PBS. Tumors were grown following subcutaneous inoculation with 5 × 10^3^ to 5 × 10^5^ cells. Tumor growth was defined at >5 mm diameter and mice were monitored for up to 6 months.

For the *in vivo* resistant model, 5 × 10^6^ cells were subcutaneously injected and tumours harvested after two weeks. Tumours were divided into pieces with diameters of 10 mm and transplanted subcutaneously. Mice were treated with cisplatin (peritoneal injection of 5 mg/kg cisplatin every four days for 4 weeks) to induce chemoresistance. As osteosarcoma cells grew rapidly *in vivo* without chemotherapy, the tumor fragments of 5mm in diameter were transplanted to set as control. Tumor volume was calculated every 3-4 days using the formula: V = length × (width)^2^/2. To examine tumorigenicity, cells dissociated from xenograft tumors (chemoresistant or vehicle) were counted by trypan blue staining, and suspended in 10μL of medium. 12 mice were randomly divided into two groups and injected with 5 × 10^4^ corresponding cells subcutaneously. When the tumors reached >5mm in diameter, they were dissociated and underwent serial transplantation using 5 × 10^4^ cells. To test the efficacy of DAPT, A total of 8 mice bearing chemoresistant xenograft tumors (at the end of the 4th week) were randomly divided into DAPT-treated and vehicle groups, with 4 mice in each group. DAPT dissolved in Dimethyl sulfoxide (DMSO) was administered by intraperitoneal injection (10 mg/kg/d) every day for 2 weeks. The vehicle group received DMSO.

### Reverse transcription quantitative real-time PCR

Total RNA was extracted using the RNeasy Plus Mini Kit and the concentration and purity was determined using an ND-1000 spectrophotometer. Reverse transcription was performed using the TaqMan Reverse Transcription Reagents. Quantitative real-time PCR reactions were set up in triplicate and performed on a 7900 PCR machine using SYBR Green PCR Master Mix. Conditions used for amplification of cDNA fragments were as follows: 95°C for 5 min, 40 cycles of amplification −95°C for 15 sec, 60°C for 1 min. Gene expression levels were calculated using the 2-ΔΔCt method and normalised to the β-actin. The gene specific primers used are listed in Supplementary Table S2.

### Western-blot analysis

Proteins were extracted with Protein Lysis Buffer. Lysates were centrifuged at 10000g at 4°C for 10min, and supernatants collected. Protein concentrations were assessed using the Bicinchoninic acid Protein Assay Kit. Cell lysates containing 40 μg protein were separated on a 10% SDS-PAGE gel and then transferred on polyvinylidene difluoride (PVDF) membranes using a Trans Blot Turbo. Membranes were blocked in a solution of Tris buffered saline containing 0.05% Tween-20 and 5% skimmed milk for 1h at room temperature. Primary antibodies were incubated overnight at 4°C. Primary antibodies used were anti-Hes1 (1:500), anti-Hey1 (1:500,) and anti-β-actin (1:2000). Horseradish peroxidase-conjugated secondary antibodies (1:3000) were incubated for 2h at room temperature. Finally, membranes were developed using an enhanced chemiluminescence substrate.

### Immunohistochemistry

Tissue sections (5 mm) were dewaxed and rehydrated. Antigen retrieval was performed by incubating slides in 10 mmol/L citric buffer (pH 6.0) and microwaving for 15 minutes. After blocking, slides were incubated with primary antibody against anti-Hes1 antibody (1:100 dilution), and anti-NICD1 antibody (1:100 dilution) overnight at 4°C, followed by biotin-conjugated secondary antibody (dilution, time), polymer horseradish peroxidase, and diaminobenzidine tetrahydroxychloride (DAB) solution.

### Immunofluorescence assay

Immunofluorescence was assessed using anti-N-cadherin antibody (1:100 dilution), anti-Stro-1 (1:200) and anti-CD117 antibody (1:200) overnight at 4°C. Secondary antibody was applied for 1 h at room temperature. Each step of the procedure was followed by a PBS wash, and the cells or tissue were counterstained with DAPI. Coverslips were mounted using Anti-fade Fluorescence Mounting Medium (Beyotime Biotechnology).

### Statistical analyses

Statistical analyses were performed using the SPSS 13.0 statistical software package. Data are expressed as the mean ± standard error of mean (SEM) of at least three independent experiments. The Student's t-test was used to compare the means of 2 groups. Where more than 3 means were compared, one-way ANOVA followed by multiple comparisons among the means was used. *P* <0.05 was considered as statistically significant.

## SUPPLEMENTARY FIGURES AND TABLES


