# Supplementary material for: Cisplatin selects for stem-like cells in osteosarcoma by activating Notch signaling
Source: Oncotarget. 2016 Apr 20;7(22):33055–68. doi: 10.18632/oncotarget.8849 (PMC5078075; doi:10.18632/oncotarget.8849)
Supplement: Supplementary file 1 [file oncotarget-07-33055-s001.pdf]

Cisplatin selects for stem-like cells in osteosarcoma by activating Notch signaling

SUPPLEMENTARY FIGURES AND TABLES

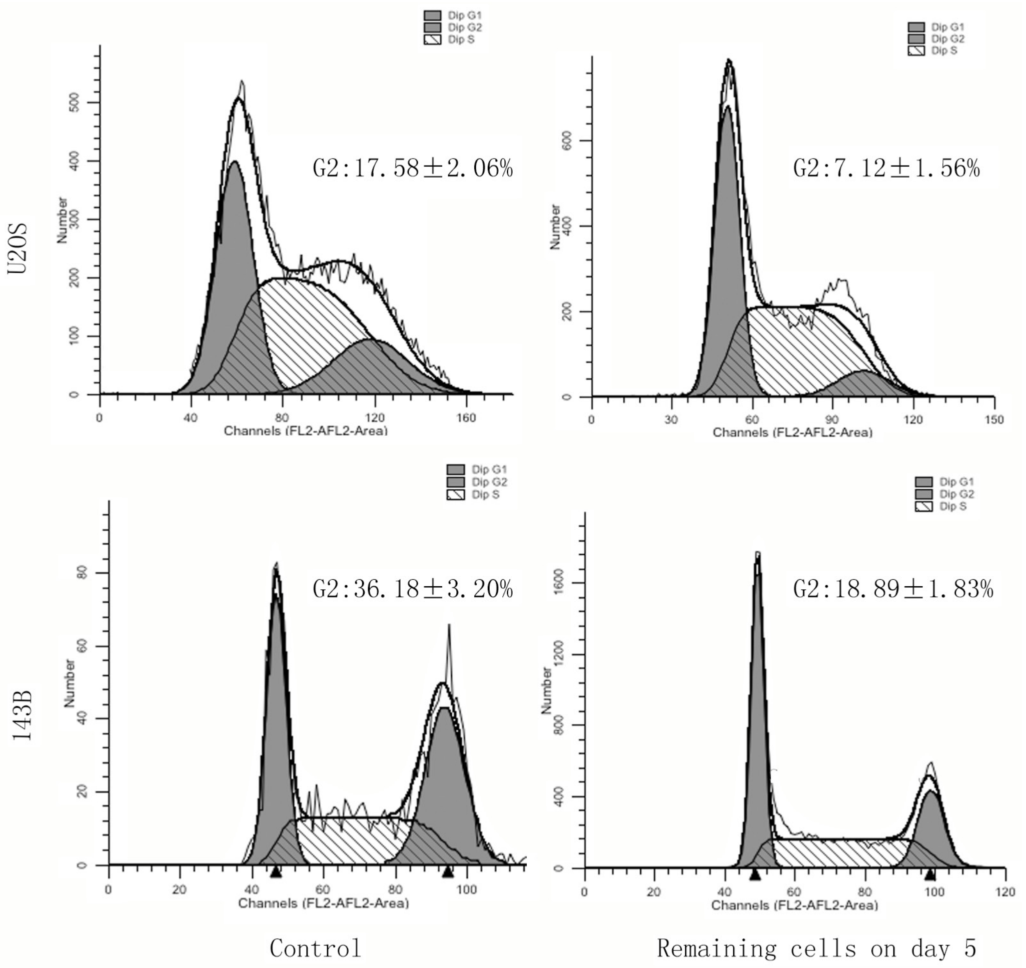

Supplementary Figure S1: The selection of cisplatin resistant osteosarcoma cells. By flow cytometry, it was demonstrated that remaining cells on day 5 had a significantly lower ratio of G2/M phase when compared with parental cells.

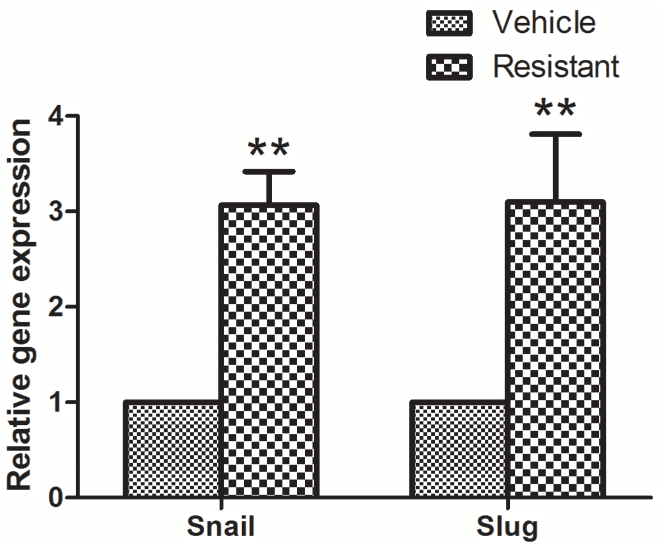

Supplementary Figure S2: Chemoresistant cells exhibit an EMT phenotype. EMT-TF genes, including snail and slug, was overexpressed in chemoresistant cells. Data were represented as mean ± SEM. $*p < 0.05$, $**p < 0.01$.

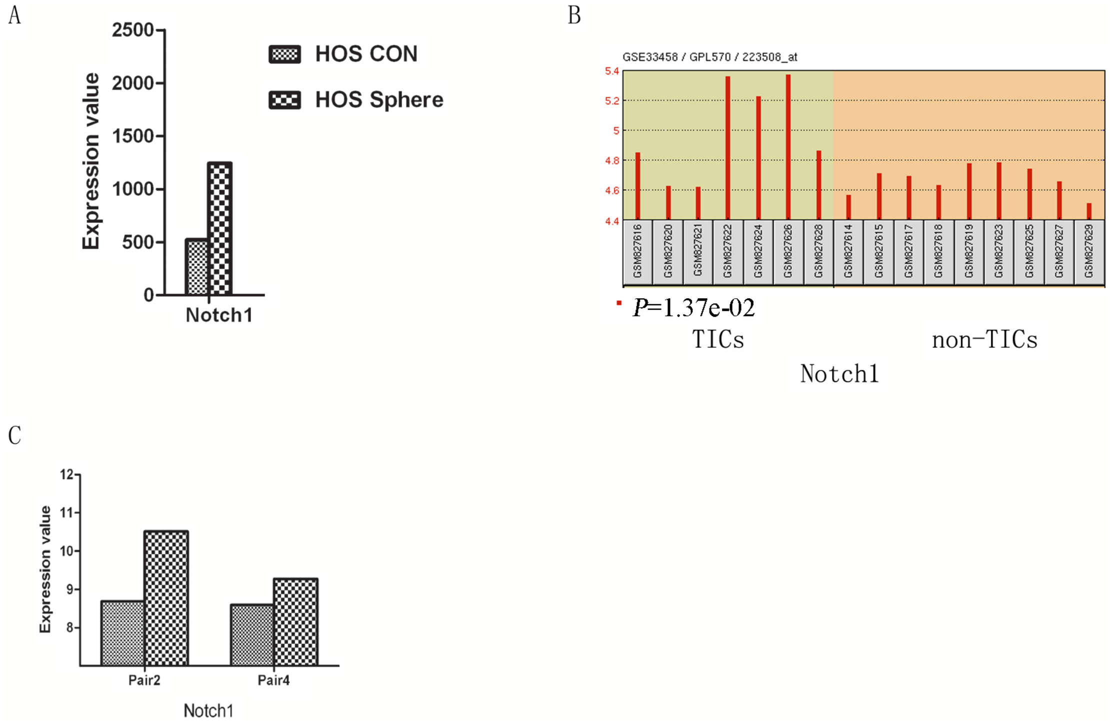

Supplementary Figure S3: Notch receptor genes expression were analyzed using publicly available datasets. A. Notch1 were upregulated in MNNG/HOS sarcospheres than adherent control. **B.** *In vivo* study showed that Notch1 were increased in osteosarcoma initiating cells. **C.** Notch1 were also increased in resection samples compared to biopsy ones.

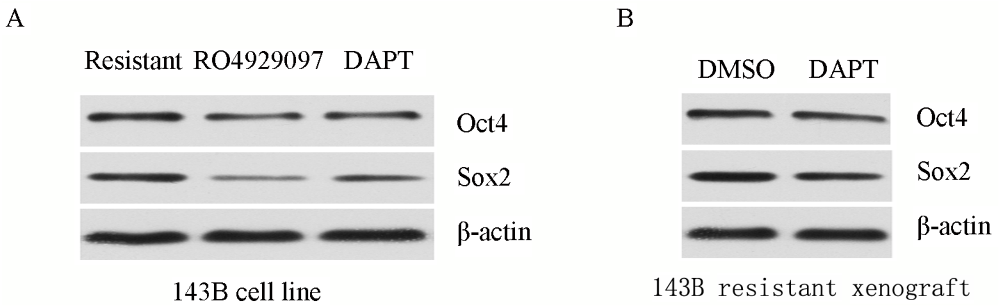

Supplementary Figure S4: The expression of stem-like cell markers were inhibited by GSIs. A. The expression of Oct4 and Sox2 were downregulated in 143B resistant cells by GSIs treatment *in vitro*. **B.** *In vivo* study showed that Oct4 and Sox2 were downregulated in 143B resistant xenografts.

Supplementary Table S1: Clinicopathological features of osteosarcoma patients

ID	Gender	Age	Site	Stage	Neoadjuvant chemotherapy
115049	M	18	Femur	N/A	Y
112193	M	21	Femur	IIB	Y
110054	M	19	Femur	IIB	Y
108741	M	19	Femur	IIB	Y
108340	M	17	Femur	IIB	Y
108007	M	9	Femur	IIB	N/A
104948	M	19	Femur	IIA	Y
103002	M	34	Femur	N/A	Y
99515	M	40	Humerus	IIB	Y
98322	M	43	Femur	N/A	Y
94024	M	19	Tibia	IIB	Y
90008	F	9	Femur	IIB	N/A
89271	M	22	Femur	IIB	Y
86268	M	13	Femur	IIB	Y
34822	F	13	Femur	IIB	Y

N/A: not available

Supplementary Table S2: Primer sequences used for Quantitative PCR

Gene	Primer sequence	
Hes1	Forward	5'-CAGATCAATGCCATGACCTACC-3'
	Reverse	5'-AGCCTCCAAACACCTTAGCC-3'
Hes5	Forward	5'-AGCCCCAAAGAGAAAAACCGACTG-3'
	Reverse	5'-TGGAGCGTCAGGAACTGCACGG-3'
Hey1	Forward	5'-CATGTCCCCAACTACATCTTCC-3'
	Reverse	5'-CCTTGCTCCATTACCTGCTTC-3'
Hey2	Forward	5'-ACCTCTCTCTTGTCCCTCTCTG-3'
	Reverse	5'-GGTTTATTGTTTGTTCCACTGC-3'
HeyL	Forward	5'-ACCGCATCAACAGTAGCCTTTCT-3'
	Reverse	5'-GCATTTTCAAGTGATCCACCGTC-3'
Oct4	Forward	5'-GAGTGAGAGGCAACCTGGAGAAT-3'
	Reverse	5'-ACCGAGGAGTACAGTGCAGTGAA-3'
Sox2	Forward	5'-TGGGTTCGGTGGTCAAGTCC-3'
	Reverse	5'-TGTGTGAGAGGGGCAGTGTG-3'
TERT	Forward	5'-GGAGCAAGTTGCAAAGCATTG-3'
	Reverse	5'-TCCCACGACGTAGTCCATGTT-3'
β-actin	Forward	5'-GTCCACCGCAAATGCTTCTA-3'
	Reverse	5'-TGCTGTCACCTTCACCGTTC-3'
